# Antibiotic prophylaxis in thyroid surgery: a preliminary multicentric italian experience

**DOI:** 10.1186/1750-1164-3-10

**Published:** 2009-08-05

**Authors:** Nicola Avenia, Alessandro Sanguinetti, Roberto Cirocchi, Giovanni Docimo, Mark Ragusa, Roberto Ruggiero, Eugenio Procaccini, Carlo Boselli, Fabio D'Ajello, Francesco Barberini, Domenico Parmeggiani, Lodovico Rosato, Francesco Sciannameo, Giorgio De Toma, Giuseppe Noya

**Affiliations:** 1 Endocrine Surgical Unit, University of Perugia, Italy; 2 General Surgical Unit, University of Perugia, Italy; 3 General Surgical Unit, Second University of Naples, Italy; 4 Thoracic Surgical Unit, University of Perugia, Italy; 5 Endocrine Surgical Unit, ASL9-H of Ivrea (Torino), Italy; 6 Department of Surgery "P. Valdoni", University of Rome, Italy

## Abstract

Post-operatory wound infections are a very uncommon finding after thyroidectomy. For these reasons international guidelines do not routinely recommend systemic antibiotic prophylaxis.

The benefits of this antibiotic prophylaxis is not supported by clinical evidence in the literature. We have conducted a multicentric randomized double-blind trial on 500 patients who had undergone thyroidectomy for goitre or thyroid carcinoma. The 500 patients enrolled in the study (mean age 47 years) were randomized in two subgroups of 250 patients. 250 patients were treated with standard antibiotic prophylaxis with sulbactam/ampicillin 1 fl (3 gr.) 30 min before surgery. No antibiotic prophylaxis was instituted in the remainder 250 patients. Our RCT showed that prophylactic antibiotic treatment is not beneficial in patients younger than eighty years old, with no concomitant metabolic, infective and hematologic disease, with no cardiac valvulopathies, not under steroidal or immunosuppressive treatment, and not severely obese. Our study should be regarded only as a preliminary RCT, and should be followed by a study in which a larger number of patients should be enrolled so that statistically significant data can be obtained.

## Introduction

The rationale of systemic antibiotic prophylaxis is to reduce the incidence of surgical infections in the surgical site [1]. Antibiotic prophylaxis should be instituted only in clean-contaminated surgical procedures or in clean surgical procedures in which an infective complication could represent an occurrence of particular severity, or in clean procedures in which prosthetic implants or other exogenous materials are being used. Antibiotic prophylaxis is not indicated in clean surgical procedures where the potential risk of side effect, bacterial and/or mycotic superinfections and the emergence of bacterial resistant strains outweighs the possible advantages. Surgical procedures are classified into four types in correlation to the increasing risk of bacterial contamination and infection [2]:

- clean surgical procedures (incidence of infections < 5%);

- clean – contaminated surgical procedures (incidence of infections < 10%);

- contaminated surgical procedures (incidence of infections about 20%);

- dirty surgical procedures (incidence of infections about 40%).

Thyroidectomy is classified amongst clean surgical procedures, those where there is no intra-operatory bacterial contamination following surgery. Post-operatory wound infections are a very uncommon finding after thyroidectomy. For these reasons international guidelines do not routinely recommend systemic antibiotic prophylaxis [1]. Although many different guidelines of the National Health Service and of Surgical Societies include these recommendations, systemic antibiotic prophylaxis is nonetheless frequently used in thyroid surgery. According to the majority of surgeons, this conduct is justified with the potential risk of infections related to the positioning of drains. The benefits of this antibiotic prophylaxis are not supported by clinical evidence in the literature.

We have conducted a multicentric randomized double-blind study on 500 patients who had undergone thyroidectomy for goitre or thyroid carcinoma. The endpoint of this study was to evaluate the benefits of antibiotic prophylaxis vs. no prophylaxis in patients undergoing thyroid surgery.

## Materials

Between January 2007 and June 2007 a multicentric randomised double blind study was conducted on 500 consecutive patients admitted to our clinical wards.

Inclusion criteria were:

- men or women between age 16 and 80;

- absence of concomitant metabolic (diabetes), infective or hematologic pathologies;

- absence of cardiac valvular pathologies;

- patients not undergoing corticosteroid or immunosuppressive treatment;

- patients in whom an aspirating drain had been positioned.

Exclusion criteria were:

- patients younger than 16 years and older than 80;

- presence of concomitant metabolic (diabetes), infective and hematologic pathologies;

- presence of severe obesity;

- ongoing corticosteroid or immunosuppressive treatment;

- presence of concomitant neoplasms;

- patients undergoing thyroidectomy and lymphadenectomy;

- patients undergoing secondary surgery in the cervical region;

- patients undergoing thyroidectomy for locally advanced tumours;

- patients undergoing thyroidectomy for autoimmune thyroiditis or Plummer adenoma;

- patients with goitres submerged in the thorax;

- patients without cervical drain.

Eligible patients were proposed to enter the RCT study upon acquisition of adequate informed consent. All patients underwent a minimum ambulatory follow up of 30 days.

## Methods

The 500 patients enrolled in this multicentric trial (mean age 47 years) were randomized in two subgroups of 250 patients. The sequence allocation generation has been formulated by a researcher of another Institution through random numbers generated by a computer; the sequence allocation concealment was actuated by means of an e-mail sent by the researcher right after enrolling the patient in the study. 250 patients were treated with standard antibiotic prophylaxis with Unasyn (sulbactam/ampicillin) 1 fl (3 gr.) 30 min before surgery. No antibiotic prophylaxis was instituted in the remaining 250 patients.

Antiseptic and aseptic measures were standardized: disinfection by means of Betadine or Hibitane (a disinfectant that does not contain iodine and that is utilized in thyroid neoplasms to avoid false results at the control scintigraphy) followed by the placement of a sterile adhesive transparent cervical film. Hemostasis was very accurate. Absorbable hemostatics (Tabotamp by Ethicon or Floseal by Baxter) were never utilized intentionally for greater homogeneity of the two subgroups. The reconstructive surgical technique was also standardized again with the intent of making the two subgroups as homogeneous as possible. Platisma reconstruction and dermal approximation was obtained with simple interrupted sutures; the skin wound was closed with metallic staples. Wounds were always dressed every third day. As a rule drains were removed on the first postoperative day; when more than 80 cc of serum was collected, the drains were left in and removed in the following days according to the clinical judgement. Within each group of patients, two subgroups were highlighted: the subgroup of patients in whom the drains were removed in the first postoperative day and the subgroup of patients in whom it was necessary to keep the drains for a longer period of time (> 1 day). The statistical analysis of the data was carried out using Review Manager 5 [3].

## Results

**Figure 1:**
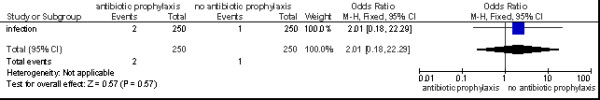
**Wound infection after thyroidectomy**.

**Figure 2:**
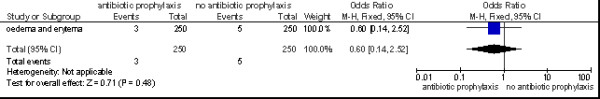
**Wound oedema and erytema after thyroidectomy**.

**Figure 3:**
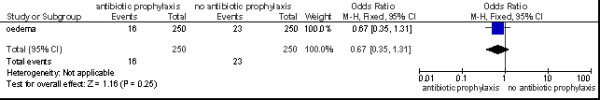
**Wound oedema after thyroidectomy**.

**Figure 4:**
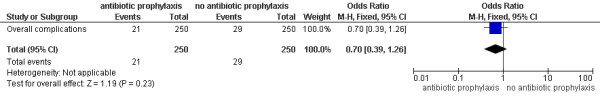
**Comprehensive analysis of all wound complications after thyroidectomy**.

Only 3 patients, two amongst those who had undergone prophylactic antibiotic treatment and one in the other group, developed an infection of the surgical wound. The infection receded after antibiotic treatment with amoxicillin/clavulanate 1 gr PO tid for 7 d. Slightly better results were obtained in the group no treated with prophylactic antibiotic treatment, although this was not statistically significant. (P = 0,57) (fig. 1). Eight patients (3 pertaining to the group of patients undergoing prophylactic antibiotic treatment and 5 to the group that did not undergo antibiotic prophylaxis) developed oedema and erythema of the surgical wound; there was no pain and hyperthermia together with these signs. No antibiotic treatment was required for their regression. The group with antibiotic prophylaxis showed better results, but this was not statistically significant. (P = 0,48) (fig. 2). Thirty-nine patients (16 pertaining to the group of patients undergoing prophylactic antibiotic treatment and 23 to the group that did not undergo antibiotic prophylaxis) developed oedema of the surgical wound; no pain and hyperthermia was evidenced along with these signs. No antibiotic treatment was required for these signs to recede. The group with antibiotic prophylaxis showed better results, but this was not statistically significant. (P = 0,25) (fig. 3). The comprehensive analysis of all the complications that were observed did not show statistically significant differences between the two groups (odds ratio (OR), 0.84; 95 percent confidence interval (CI), 0.39–1.26; P = 0,23) (fig. 4).

**Figure 5:**
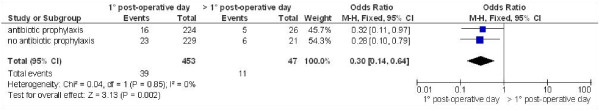
**Comprehensive analysis of all wound complications after thyroidectomy: patients in whom the drain was removed in the first post-operative day vs those in whom it was removed in the following days**.

**Figure 6:**
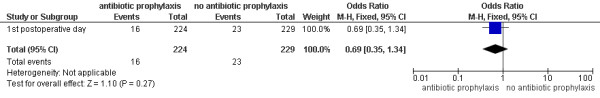
**Comprehensive analysis of all wound complications after thyroidectomy in patients in whom the drain was removed in the first post-operative day: antibiotic prophylaxis vs no antibiotic prophylaxis**.

**Figure 7:**
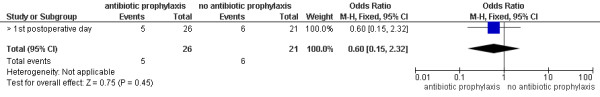
**Comprehensive analysis of all wound complications after thyroidectomy in patients in whom the drain was removed after the first post-operative day: antibiotic prophylaxis vs no antibiotic prophylaxis**.

We performed a subgroup analysis between patients in whom the drain was removed in the first post-operative day and those in whom it was removed in the following days. In 47 patients only it was necessary to keep the drain beyond the first post-operative day. Patients from whom the drain was removed in the first postoperative day showed a slightly inferior incidence of wound complications related to infective occurrence (lower incidence of oedema, erythema or infection of the surgical wound) (P = 0,002) (fig. 5). No significant advantage was evidenced with prophylactic antibiotic treatment in patients in whom the drain was removed on the 1st postoperative day (P = 0,27) (fig. 6). A significative advantage was not substantiated with prophylactic antibiotic treatment even in patients in whom the drain was kept after the 1st postoperative day (P = 0,45) (fig. 7).

## Discussion

Thyroid surgery is classified as a clean procedure and is associated with a low incidence of wound infections. (0,3%) [4]. Although the majority of international guidelines do not recommend the use of systemic prophylactic antibiotic treatment, this practice is adopted sporadically in some nations and routinely in others [5]. An audit carried out by the British Association of Endocrine Surgery (BAES) in England and Ireland showed that 9% of patients received routine antibiotic prophylaxis, 16% in selected cases and 75% did not receive it [6]. In an Italian retrospective study carried out by Rosato on 14.394 patients, it was evidenced that 50% of surgeons use antibiotic prophylaxis, 17% antibiotic therapy and 33% neither prophylaxis nor therapy [4]. The Scottish Intercollegiate Guidelines network (SIGN) does not advocate antibiotic prophylaxis for benign pathologies regarding it an opportunity to be reserved for selected cases of malignancies [7]. The BAES and the Royal College of Physicians Thyroid Cancer do not list antibiotic prophylaxis amongst the recommendations to follow for thyroidectomy [8,9]. Antibiotic prophylaxis does not ward off the development of infective complications of the surgical wound [4]. The occurrence of severe cervical infections after thyroid surgery is an extremely rare event with an extremely high inherent mortality rate; however the occurrence of these infections, often cellulites with fulminating Streptococcal sepsis cannot be avoided with antibiotic prophylaxis [6].

In common clinical practice it is believed that the use of drains after thyroidectomy represents a risk factor for the development of infective complications of the surgical wound. This notion is not supported by clinical data, whereas conflicting evidence was presented recently in a systematic review by Samraj e Gurusamy [10]. This review of the literature has evidenced 5 RCT in which 337 patients with drains were compared with 350 patients without drains following thyroidectomy; the statistical analysis failed to evidence that patients with drains show a significant incidence of infections of the surgical wound (P = 0,41). Therefore antibiotic prophylaxis does not seem to be beneficial in all patients with drains [11].

## Conclusion

Our RCT showed that prophylactic antibiotic treatment is not beneficial in patients younger than eighty years old, with no concomitant metabolic, infective or hematologic disease, with no cardiac valvulopathies, not receiving steroidal or immunosuppressive treatment, and not severely obese. Thyroidectomies with associated lymphadenectomy, secondary surgery, excision of locally advanced disease, or of autoimmune thyroiditis and Plummer adenoma were excluded from this analysis. Drains did not represent a risk factor for the development of infections of the surgical wound even in the group of patients not undergoing antibiotic prophylaxis. Our study should be regarded only as a preliminary RCT, and should be followed by a study in which a larger number of patients should be enrolled so that statistically significant data can be obtained.

## Competing interests

The Authors state that none of the authors involved in the manuscript preparation has any conflicts of interest towards the manuscript itself, neither financial nor moral conflicts. Besides none of the authors received support in the form of grants, equipment, and/or pharmaceutical items.

## Authors' contributions

All authors contributed equally to this work. RC participated in the design of the study and performed the statistical analysis. All authors read and approved the final manuscript.
